# Electroacupuncture Reduces Hyperalgesia after Injections of Acidic Saline in Rats

**DOI:** 10.1155/2014/485043

**Published:** 2014-03-19

**Authors:** Leonardo Yung dos Santos Maciel, Kamilla Mayara Lucas da Cruz, Ariane Martins de Araujo, Zak Moreira de Andrade Silva, Daniel Badauê-Passos, Valter Joviniano Santana-Filho, Josimari Melo DeSantana

**Affiliations:** ^1^Graduate Program in Health Science, Federal University of Sergipe, Aracaju, SE, Brazil; ^2^Graduate Program in Physiological Science, Federal University of Sergipe, Aracaju, SE, Brazil; ^3^Department of Physical Therapy, Federal University of Sergipe, Aracaju, SE, Brazil; ^4^Department of Physiology, Federal University of Sergipe, Aracaju, SE, Brazil

## Abstract

*Background*. Injections of acidic saline into the gastrocnemius muscle in rats produce a bilateral long-lasting hyperalgesia similar to fibromyalgia in humans. No previous study investigated the effect of electroacupuncture (EA) on this acidic saline model. This study aimed to identify the effects of EA in the hyperalgesia produced by repeated intramuscular injections of acidic saline. *Methods*. Rats were divided into four groups (*n* = 6, each group): control, acupuncture, EA 15 Hz, and 100 Hz. Left gastrocnemius muscle was injected with 100 **μ**L of pH 4.0 sterile saline twice five days apart. EA, acupuncture, or control therapy was daily administered (20 min) for 5 consecutive days under anesthesia. Needles were placed in the St36 and Sp6 acupoints. The assessment of secondary mechanical hyperalgesia, thermal hyperalgesia, and motor performance was performed before injections and before and after the treatment performed on each day. The paw withdrawal threshold was tested using the nonparametric Kruskal-Wallis test and differences within the group Wilcoxon Matched Pairs. The latency and motor performance were tested for ANOVA parametric test for independent measures, and for differences in the group, we used *t*-test for paired samples. Post hoc Tukey test was used for multiple corrections. *P* values **less than 0.05 were considered statistically significant. *Results*. Indicate that there was a significant reduction of mechanical withdrawal threshold and paw withdrawal latency 24 hours following the second injection. Moreover, mechanical and thermal hyperalgesia were significantly reversed by EA 15, 100 Hz, and acupuncture. *Conclusions*. The results suggest that EA high and low frequency as well as acupuncture are effective in reducing hyperalgesia in chronic muscle pain model.

## 1. Introduction

Musculoskeletal pain is common and costs billions of dollars in health care and lost wages [1]. Chronic musculoskeletal pain syndromes such as fibromyalgia and myofascial pain syndrome can be disabling and difficult to treat [2]. Focusing on these issues, a model of long-lasting mechanical hypersensitivity induced by 2 intramuscular injections of acidic (pH 4.0) saline [3] was developed. This procedure produces a bilateral muscle and cutaneous mechanical hypersensitivity and increased visceral sensitivity [3, 4].

Only 5 studies have shown in this model of pain after double injection of acidic saline some treatment and two nonpharmacological treatments [5, 6], where also the interaction of the opioid system in effect after physical activity was proved [5] and three studies in which pharmacological treatments reversed the hyperalgesia [4, 7, 8]. The efficacy of electroacupuncture based analgesia is well documented, and some trials have demonstrated that electroacupuncture reduces the need of postoperative consumption of analgesic drugs [9, 10]. Other studies have shown that EA is effective in treating migraine crisis [11] and orofacial pain [12] without the use of pharmacological agents. The promotion of the EA analgesic effect in animal models of acute or chronic pain is well documented in the literature [13–24] demonstrating the potential of this therapy.

Studies have shown the activation of descending inhibitory ways, descending opioid system after stimulation by EA [16, 25, 26], an increase in *β*-endorphin levels of plasma [24, 27], and brain [19] interactions with the GABAergic system [28], activation of spinal muscarinic receptors [29, 30], and involvement of the serotonergic system [16] in other animal models of hyperalgesia. Concerning the best frequency for treatment with EA, the literature is unclear about the most efficient way. Some studies showed better results with low frequency in an animal model [13, 17] while in other studies high-frequency EA was more effective [31].

The objective of the present study was to determine the effect of electroacupuncture and acupuncture to reduce antinociception in the animal model of fibromyalgia.

## 2. Methods

All experiments were approved by the Animal Care and Use Committee at the Federal University of Sergipe and are in accordance with the guidelines of the International Association for the Study of Pain on use of laboratory animals. Adult male Wistar rats (*n* = 24, 250–300 g) were used for this study.

### 2.1. Muscle-Induced Hyperalgesia

Immediately after baseline behavioral measurements as described below, rats were anesthetized with isoflurane (2% to 5%) and injected with 100 mL of pH 4.0 sterile saline into the gastrocnemius muscle of the left hind limb on Day 0 (injection 1) and again on Day 5 (injection 2). This procedure causes a bilateral mechanical hypersensitivity of the muscle and paw that lasts up to 4 weeks [3, 4, 32].

### 2.2. Secondary Mechanical Hyperalgesia

Rats were tested for paw withdrawal threshold with von Frey filaments applied to the plantar surface of the paw. Initially, the animals were acclimated two consecutive days for mechanical threshold measurement before starting the experiment protocol. Animals were conducted to behavioral room for 30 minutes and then placed in transparent Lucite cubicles on a wire mesh elevated plate and acclimated for another 30 minutes each day. Then, the animals were again acclimated within their home cages in the behavior room for 30 minutes and placed in the cubicles for 30 minutes every day before testing. A series of filaments with increasing bending forces (11,8 to 190,9 mN) were applied twice on the plantar surface of the hind paw until the rat withdrew from the stimulus. The lowest force at which the rat withdrew its paw from 1 of 2 applications was recorded as the paw withdrawal threshold. A decrease in paw withdrawal threshold was interpreted as cutaneous hypersensitivity. This testing method has shown significant test-retest reliability and even injection of acidic saline was applied to the unilateral muscle; this test is used to capture very sensitization, since this promotes an animal model with central sensitization bilateral involvement [3].

### 2.3. Thermal Hyperalgesia

The test was performed using the Tail Flick apparatus where the animals were placed in clear acrylic structure in which only the tail faced outside, supported on a metal structure heated to 50°C over a period of 20 seconds. The time the rat took to remove the tail of the blade indicated time tolerance of the animal to the heat generated in the blade.

Thermal hyperalgesia test was performed in the next moment of the completion of the measurement of mechanical threshold and the engine performance test at the following times: prior to the first and second acid saline injection, 24 hours after the second injection of acidic saline, and immediately before and after each therapeutic application of appeal on five consecutive days of treatment after induction of muscle pain.

### 2.4. Motor Performance

The motor effects promoted by electroacupunture or acupuncture in rats were tested by using Rota-rod treadmill. Specifically, the animals were placed on the Rota-rod running at speed with a gradual increase from 1 to 18 rotations per minute (rpm) for 120 seconds and maintained for another 30 seconds at 18 rpm [3].

The motor performance test was conducted at the same time as the measurement of mechanical threshold and thermal test at the following times: before the first and second acidic saline injection, 24 hours after the second injection of acidic saline, and immediately before and after application of each therapeutic resource for the five consecutive days of treatment after induction of muscle pain.

### 2.5. Acupuncture

**Figure 1:**
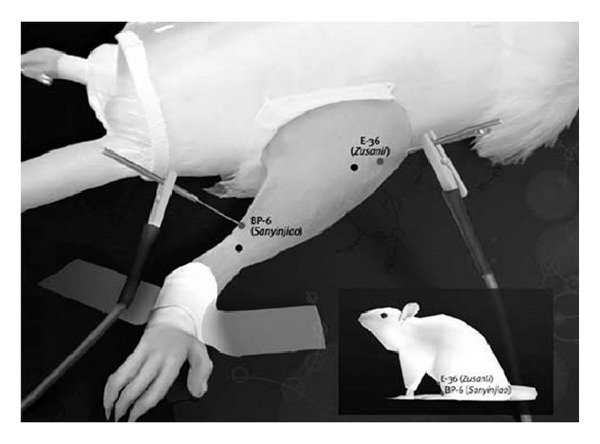
Acupuncture points St36 and Sp6.

Stainless steel needles (30 × 0.25 mm) were inserted into the acupoint St36, located in the anterior tibial muscle, 10 mm distal to the knee joint, and into the acupoint Sp6, located above the tip of the medial malleolus (Figure 1), for 20 minutes; independent needles were stimulated manually every 5 minutes.

### 2.6. Electroacupuncture Stimulation

Stainless steel needles (30 × 0.25 mm) were inserted into the acupoint St36, located in the anterior tibial muscle, 10 mm distal to the knee joint, and into the acupoint Sp6, located above the tip of the medial malleolus (Figure 1), when both acupuncture or electroacupuncture were used. For the electroacupuncture treatments, the needles were connected to an electronic pulse generator output (NKL Portable EL 608, Brazil), which produces a bipolar and asymmetric square wave. The frequencies tested were 15 Hz and 100 Hz, and the duration of stimulation was 20 min for both frequencies. Stimulus intensity was maintained at a sensory threshold, just below a detectable muscle twitch, in order to mimic the intensity used in clinical practice as closely as possible.

All animals were stimulated under isoflurane anesthesia. Control animals were anesthetized with isoflurane for the same amount of time, but no needle was used nor electrical current was delivered. We chose the model under anesthesia, because the model does not use the anesthetic effect causing many injuries in animals, thus hindering the real perception of treatment effect.

### 2.7. Experimental Design

**Figure 2:**
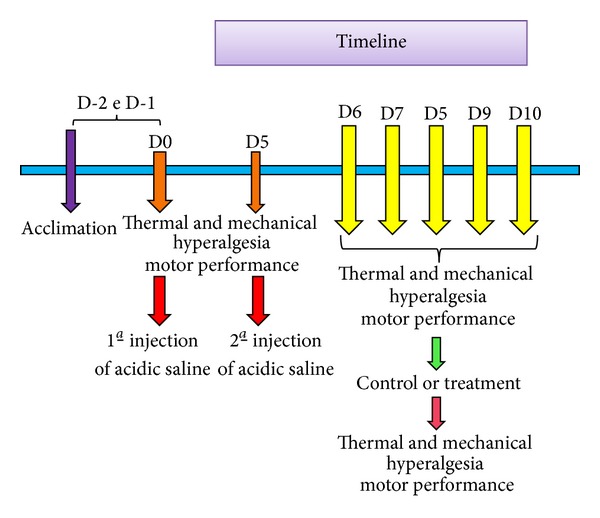
Timeline of experimental protocol.

All animals were acclimated two consecutive days before starting the experimentation. Mechanical paw withdrawal threshold, thermal hyperalgesia, and motor performance were measured before both first and second injection of acidic saline and again immediately before and after all interventions during five consecutive days of treatment. The timeline for the experiment is presented in Figure 2.

### 2.8. Statistical Analysis

The paw withdrawal threshold was tested for differences between treatment groups by using the nonparametric Kruskal-Wallis test and differences within the group Wilcoxon Matched Pairs. The latency and motor performance were tested for differences between treatment groups by ANOVA parametric test for independent measures, and for differences in the group, we used *t*-test for paired samples. Post hoc Tukey test was used for multiple corrections. *P* values less than 0.05 were considered statistically significant.

## 3. Results and Discussion

### 3.1. Paw Withdrawal Threshold

**Figure 3:**
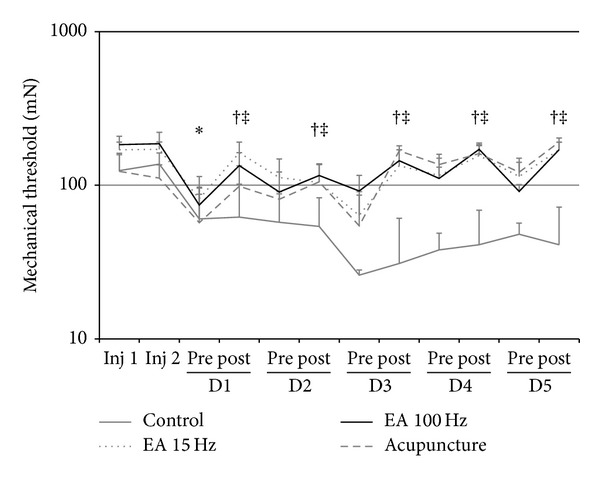
Line graph representing the mechanical threshold (in mN) of ipsilateral paw withdrawal groups 15 Hz and 100 Hz electroacupuncture, acupuncture, and control. Values are presented as mean ± standard error of mean. Inj: injection; 1: before; 2: after. *P < 0.05 compared to control group and the pretreatment. Kruskal-Wallis test for independent measures, adjusted by the Tukey test, and Wilcoxon Matched Pairs test for paired measurements.

All groups showed a significant reduction in bilateral mechanical withdrawal threshold of the paw (*P* < 0.03) 24 h after the second injection of acidic saline (*P* < 0.05). However, there was a significant reversal of mechanical hyperalgesia in the groups treated with both EA (100 Hz and 15 Hz) and acupuncture for five consecutive days when compared to control (*P* < 0.05, Figure 3).

### 3.2. Thermal Hyperalgesia

**Figure 4:**
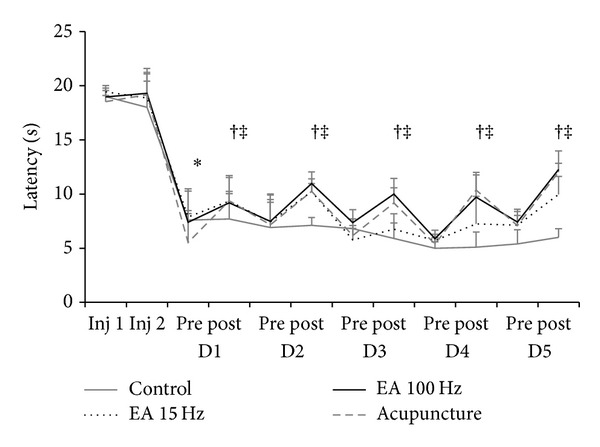
Line graph representing latency (in seconds) to withdraw the tail ipsilateral groups 15 Hz and 100 Hz electroacupuncture, acupuncture, and control. Values are presented as mean ± standard error of mean. Inj: injection; 1: before; 2: after. *P < 0.05 compared to control group and the pretreatment. Measures: ANOVA test for paired and independent measures, adjusted by the Tukey test.

Twenty-four hours after the induction of muscle hyperalgesia by the second injection of acidic saline, the latency (*P* < 0.001) was significantly reduced in the tail. After treatment there was a significant reversal withdrawal threshold in all treated groups compared with the control group (*P* < 0.05) (Figure 4).

### 3.3. Motor Performance

**Figure 5:**
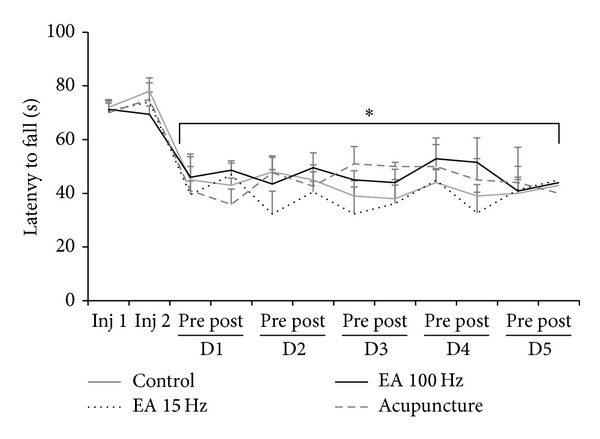
Line graph representing the motor performance (in seconds) of the animals in groups 15 Hz and 100 Hz electroacupuncture, acupuncture, and control. Values are presented as mean ± standard error of mean. Inj: injection; 1: before; 2: after. *P < 0.05 compared to control group and the pretreatment. Measures: ANOVA test for paired and independent measures, adjusted by the Tukey test.

There was a significant reduction of the time to fall in a Rota-rod apparatus in all groups 24 h following the second injection of acidic saline (*P* < 0.05), which was kept over the time of experimentation through five days of treatment (Figure 5).

The results of this study demonstrate that both electroacupuncture and acupuncture reduced the mechanical hyperalgesia and thermal hyperalgesia following administration of associated double intramuscular injection of acidic saline which generates muscle hyperalgesia that simulated experimentally fibromyalgia syndrome. In parallel, the motor performance was optimized in groups that received electrical stimulation.

To our knowledge, this is the first work of experimental animal study in rats investigating the effects of both electroacupuncture and acupuncture on an animal model of fibromyalgia. However, similar to our findings, some previous experimental studies have demonstrated the EA effect using different animal models: reduction of mechanical hyperalgesia, such as in models of inflammatory pain induced by carrageenan [20, 21]; nerve growth factor [17] or Freund's adjuvant [14, 16, 18, 22, 23, 33], in models of neuropathic pain after spinal section between S3 and S4 [13] and ligation of the anterior tibial and sural nerve [34]; pain ankle sprain [15]; ovariectomy in female dogs [24] and in rats [27] and in animal models of cancer induction [19]. Acupuncture has shown antihyperalgesic effect in neuropathic model [35] of pain induced by formalin injection [36]; however, there was no significant difference between acupuncture and EA in inflammatory pain model induced by Freund's adjuvant [37] and carrageenan [38].

In the present study, EA and acupuncture were able to promote reduction of the thermal hyperalgesia in this model of noninflammatory muscle pain. Some studies have demonstrated the EA-induced antihyperalgesia caused by EA in models of inflammatory pain by injection of Freund's adjuvant [16, 18, 33] or carrageenan [39], neuropathic pain by section between S1 and S2 [28, 30], and through the tail flick test in mice to verify the effectiveness of different frequencies of EA [40]. However, in studies of inflammatory pain induced by administration of Freund's adjuvant [14] and neuropathic pain [39], there were no significant changes in thermal hyperalgesia between groups treated with control group.

Our findings showed that in the present study, the motor performance of rats showed no change in the latency to fall compared to control animals. Except for the decrease 24 hours after the second injection, when the muscle hyperalgesia is supposed to be maximum and motor performance fell. Firstly, This suggests that reversal of hyperalgesia produced by EA in both frequency bandies, as well as acupuncture, is actually assigned antihyperalgesic action of stimulation, as sedative or muscle relaxants could change motor performance after treatment. Moreover, it seems that the development of muscle widespread hyperalgesia in this experimental model was responsible for impairing studies that correlate the EA and motor performance in models of hyperalgesia in rats, which are scarce. On the other hand Jia et al. [41] showed significant improvement in motor efficiency and coordination in mice after induction of Parkinson's disease by unilateral section of the medial forebrain bundle treated with high- and low-frequency electroacupuncture, but only the high frequency (100 Hz) demonstrated an improvement in this model for coordination and motor performance, which was not observed in our study.

After repeated use of electroacupuncture, we observed maintenance of analgesic efficacy of both low and high frequency, although some studies suggest the development of analgesic tolerance and the possible involvement of the opioidergic in mediating the effect of EA in both frequency bands. Similar to our findings, consecutive applications of EA for three days in a model of visceral hypersensitivity in mice did not develop tolerance to the analgesic treatment with electroacupuncture [42]. However, some studies have demonstrated the participation of opioid system in the antihyperalgesia of electroacupuncture promoted by the application through the administration of blockers of *μ* and *δ* opioid receptors [8, 16, 19–21, 27, 31, 33, 36, 43]. Other studies also confirmed the action of *β*-endorphin as evidenced, an increase in plasma after application of electroacupuncture in the postoperative ovariectomy in female dogs [24] and rats [27]. The increase in blood and brain *β*-endorphin has been demonstrated in a model of cancer induction in rats [19]. Although with these data we can not affirm the involvement of the opioid system, we believe this possibility, and yet we did not observe the effect of electroacupuncture reduced after 5 days of treatment in this animal model.

The involvement of the GABAergic system was demonstrated by Park et al. [28] in a model of neuropathic pain in rats; the blockade of GABA (A) and GABA (B) reversed the antihyperalgesic effect after stimulation of low-frequency EA (2 Hz). In parallel, 2 Hz EA operates in spinal muscarinic receptors, after administration of atropine that reversed the analgesic effects produced by EA [29, 30]. The serotonergic and glutamatergic systems also had their shares in mediating the effect of EA evidenced in previous studies. In animal model of hyperalgesia induced with Freund's adjuvant, after the application of EA was producing of catecholamines and serotonin, demonstrating the activation of these pathways in controlling pain [16]. Also, EA showed a decrease in phosphorylation of the subunits of spinal NMDA (N-methyl-D-aspartate), also demonstrating the involvement of these structures in the process of stimulation of EA analgesia [42–45]. Our data do not allow us to say exactly which pain inhibition system is acting.

Only five previous studies have investigated the anti-hyperalgesic effects of therapeutic strategies in experimental model of fibromyalgia in rats, one with pharmacological treatment and the others with non-pharmacological techniques. The reduction of hyperalgesia produced by morphine, SNC80, damge, and selective opioid receptor agonists was prevented by blocking opioid receptors *μ* and *δ* but not *κ*. Therefore, activation of spinal *μ* opioid receptors and *δ* reduces mechanical hyperalgesia following repeated intramuscular injection of acidic saline [7]. Pregabalin reduces mechanical hyperalgesia, but there was motor impairment in higher dosages [4] and the combination of tramadol and milnacipran enhances the antihyperalgesia in this animal model of FM [8]. The reversal of secondary mechanical hyperalgesia after physical exercises of low intensity [5, 6] was proven in the same animal model used by us and the action of the opioid system stimulated by exercise was shown when receptor blockade by naloxone interrupted the analgesic effect.

Clinically, the use of EA in patients with fibromyalgia was investigated in only one controlled clinical trial. In this study, fibromyalgia patients were treated with six sessions of EA for three weeks, using frequency that varied between 1 and 99 Hz, with intensity-level motor contraction. Compared to the control group, subjects treated showed significant analgesic effects, reducing the intensity and distribution of pain and analgesic consumption, increased pressure pain threshold, and improved quality of sleep [46].

Our work got some similar results in behavioral tests to those found in previous studies that showed the involvement of the opioid system in the reversal of hyperalgesia in animal models of pain through the use of electroacupuncture, which for us is a possibility, but the effect of treatment developed in our work did not lose its effectiveness even after 5 consecutive days, so we did not develop tolerance to treatment. We are doing some work to elucidate the possible mechanisms of acupuncture and electroacupuncture analgesia by this animal model both intrathecal and intracerebral level through blockers naloxone and naltrindole.

## 4. Conclusions

All our data suggested that EA high and low frequency and acupuncture have the ability to reverse the mechanical and thermal hyperalgesia in animal models of chronic muscle widespread pain, diffuse, and bilateral noninflammatory produced by the double injection of saline acidic (pH 4.0). With regard to motor performance, the treatment groups (acupuncture, EA 15 Hz and 100 Hz) showed no significant difference in time spent in the Rota-rod compared to control. Studies are being conducted to better understand the mechanisms of action of EA in this animal model of hyperalgesia muscle pain, especially interactions with the opioid system, as well as other central and peripheral mechanisms.

## References

[1] Yelin E, Callahan LF (1995). The economic cost and social and psychological impact of musculoskeletal conditions. *Arthritis and Rheumatism*.

[2] McCain GA (1996). A cost-effective approach to the diagnosis and treatment of fibromyalgia. *Rheumatic Disease Clinics of North America*.

[3] Sluka KA, Kalra A, Moore SA (2001). Unilateral intramuscular injections of acidic saline produce a bilateral, long-lasting hyperalgesia. *Muscle & Nerve*.

[4] Yokoyama T, Maeda Y, Audette KM, Sluka KA (2007). Pregabalin reduces muscle and cutaneous hyperalgesia in two models of chronic muscle pain in rats. *Journal of Pain*.

[5] Hoeger Bement MK, Sluka KA (2005). Low-intensity exercise reverses chronic muscle pain in the rat in a naloxone-dependent manner. *Archives of Physical Medicine and Rehabilitation*.

[6] Sharma NK, Ryals JM, Gajewski BJ, Wright DE (2010). Aerobic exercise alters analgesia and neurotrophin-3 synthesis in an animal model of chronic widespread pain. *Physical Therapy*.

[7] Sluka KA, Rohlwing JJ, Bussey RA, Eikenberry SA, Wilken JM (2002). Chronic muscle pain induced by repeated acid injection is reversed by spinally administered *μ*- and *δ*-, but not *κ*-, opioid receptor agonists. *Journal of Pharmacology and Experimental Therapeutics*.

[8] Kim S-H, Song J, Mun H, Keon UP (2009). Effect of the combined use of tramadol and milnacipran on pain threshold in an animal model of fibromyalgia. *Korean Journal of Internal Medicine*.

[9] Shankar N, Varshney A, Bhattacharya A, Sharma KN (1996). Electroacupuncture, morphine and clonidine: a comparative study of analgesic effects. *Indian Journal of Physiology and Pharmacology*.

[10] Zhang R-X, Lao L, Wang X, Ren K, Berman BB (2004). Electroacupuncture combined with indomethacin enhances antihyperalgesia in inflammatory rats. *Pharmacology Biochemistry and Behavior*.

[11] Zhao C-H, Stillman MJ, Rozen TD (2005). Traditional and evidence-based acupuncture in headache management: theory, mechanism, and practice. *Headache*.

[12] Goddard G (2005). Short term pain reduction with acupuncture treatment for chronic orofacial pain patients. *Medical Science Monitor*.

[13] Hwang BG, Min B-I, Kim JH, Na HS, Park DS (2002). Effects of electroacupuncture on the mechanical allodynia in the rat model of neuropathic pain. *Neuroscience Letters*.

[14] Huang C, Hu Z-P, Long H, Shi Y-S, Han J-S, Wan Y (2004). Attenuation of mechanical but not thermal hyperalgesia by electroacupuncture with the involvement of opioids in rat model of chronic inflammatory pain. *Brain Research Bulletin*.

[15] Tae SH (2007). The effect of 2 Hz and 100 Hz electrical stimulation of acupoint on ankle sprain in rats. *Journal of Korean Medical Science*.

[16] Li A, Wang Y, Xin J (2007). Electroacupuncture suppresses hyperalgesia and spinal Fos expression by activating the descending inhibitory system. *Brain Research*.

[17] Aloe L, Manni L (2009). Low-frequency electro-acupuncture reduces the nociceptive response and the pain mediator enhancement induced by nerve growth factor. *Neuroscience Letters*.

[18] Chen L, Zhang J, Li F (2009). Endogenous anandamide and cannabinoid receptor-2 contribute to electroacupuncture analgesia in rats. *Journal of Pain*.

[19] Lee H-J, Lee J-H, Lee E-O (2009). Substance P and beta endorphin mediate electroacupuncture induced analgesic activity in mouse cancer pain model. *Acupuncture & Electro-Therapeutics Research*.

[20] Taguchi R, Taguchi T, Kitakoji H (2010). Involvement of peripheral opioid receptors in electroacupuncture analgesia for carrageenan-induced hyperalgesia. *Brain Research*.

[21] Yang EJ, Koo ST, Kim YS (2011). Contralateral electroacupuncture pretreatment suppresses carrageenan-induced inflammatory pain via the opioid-mu receptor. *Rheumatology International*.

[22] Zhang Y, Meng X, Li A (2011). Acupuncture alleviates the affective dimension of pain in a rat model of inflammatory hyperalgesia. *Neurochemical Research*.

[23] Zhang Y, Li A, Xin J (2011). Involvement of spinal serotonin receptors in electroacupuncture anti-hyperalgesia in an inflammatory pain rat model. *Neurochemical Research*.

[24] Groppetti D, Pecile AM, Sacerdote P, Bronzo V, Ravasio G (2011). Effectiveness of electroacupuncture analgesia compared with opioid administration in a dog model: a pilot study. *British Journal of Anaesthesia*.

[25] Kim H-W, Uh D-K, Yoon S-Y (2008). Low-frequency electroacupuncture suppresses carrageenan-induced paw inflammation in mice via sympathetic post-ganglionic neurons, while high-frequency EA suppression is mediated by the sympathoadrenal medullary axis. *Brain Research Bulletin*.

[26] Pereira KS (2008). Preemptive analgesia with peripheral neural stimulation (acupuncture) for patients of surgery of third molar inferiors with osteotomy. *Revista Dor*.

[27] Liu J-L, Chen S-P, Gao Y-H, Meng F-Y, Wang S-B, Wang J-Y (2010). Effects of repeated electroacupuncture on *β*-endorphin and adrencorticotropic hormone levels in the hypothalamus and pituitary in rats with chronic pain and ovariectomy. *Chinese Journal of Integrative Medicine*.

[28] Park J-H, Han J-B, Kim S-K (2010). Spinal GABA receptors mediate the suppressive effect of electroacupuncture on cold allodynia in rats. *Brain Research*.

[29] Baek YH, Do YC, Hyung IY, Park DS (2005). Analgesic effect of electroacupuncture on inflammatory pain in the rat model of collagen-induced arthritis: mediation by cholinergic and serotonergic receptors. *Brain Research*.

[30] Park JH, Kim SK, Kim HN (2009). Spinal cholinergic mechanism of the relieving effects of electroacupuncture on cold and warm allodynia in a rat model of neuropathic pain. *Journal of Physiological Sciences*.

[31] Han J-S (2004). Acupuncture and endorphins. *Neuroscience Letters*.

[32] Da Silva LF, DeSantana JM, Sluka KA (2010). Activation of NMDA receptors in the brainstem, rostral ventromedial medulla, and nucleus reticularis gigantocellularis mediates mechanical hyperalgesia produced by repeated intramuscular injections of acidic saline in rats. *Journal of Pain*.

[33] Zhang GG, Yu C, Lee W, Lao L, Ren K, Berman BM (2005). Involvement of peripheral opioid mechanisms in electroacupuncture analgesia. *Explore*.

[34] Cha MH, Bai SJ, Lee KH (2010). Acute electroacupuncture inhibits nitric oxide synthase expression in the spinal cord of neuropathic rats. *Neurological Research*.

[35] Cidral-Filho FJ, da Silva MD, Moré AOO, Córdova MM, Werner MF, Santos ARS (2011). Manual acupuncture inhibits mechanical hypersensitivity induced by spinal nerve ligation in rats. *Neuroscience*.

[36] Kim GH, Yeom M, Yin CS (2010). Acupuncture manipulation enhances anti-nociceptive effect on formalin-induced pain in rats. *Neurological Research*.

[37] Huang H, Zhan R, Yu X-J, Zhang D, Li W-M, Ding G-H (2009). Effects of acupoint-nerve block on mast cell activity, manual acupuncture- and electroacupuncture-induced analgesia in adjuvant arthritis rats. *Zhen ci yan jiu*.

[38] Oh JH, Bai SJ, Cho Z-H (2006). Pain-relieving effects of acupuncture and electroacupuncture in an animal model of arthritic pain. *International Journal of Neuroscience*.

[39] Ma C, Li C-X, Yi J-L, Yan L-P (2008). Effects of electroacupuncture on glutamate and aspartic acid contents in the dorsal root ganglion and spinal cord in rats with neuropathic pain. *Zhen ci yan jiu*.

[40] Silva JRT, Silva ML, Prado WA (2011). Analgesia induced by 2- or 100-Hz electroacupuncture in the rat tail-flick test depends on the activation of different descending pain inhibitory mechanisms. *Journal of Pain*.

[41] Jia J, Li B, Sun Z-L, Yu F, Wang X, Wang X-M (2010). Electro-acupuncture stimulation acts on the basal ganglia output pathway to ameliorate motor impairment in parkinsonian model rats. *Behavioral Neuroscience*.

[42] Tian S-L, Wang X-Y, Ding G-H (2008). Repeated electro-acupuncture attenuates chronic visceral hypersensitivity and spinal cord NMDA receptor phosphorylation in a rat irritable bowel syndrome model. *Life Sciences*.

[43] Chen W, Yang J, Shi J, Liu X, Guan X (2003). Effects of electroacupuncture on the pain threshold and the NMDA R1 mRNA in DRG on neuropathic pain rats. *Journal of Huazhong University of Science and Technology*.

[44] Almeida RT, Perez AC, Francischi JN, Castro MS, Duarte IDG (2008). Opioidergic orofacial antinociception induced by electroacupuncture at acupoint St36. *Brazilian Journal of Medical and Biological Research*.

[45] Jung TG, Lee JH, Lee IS, Choi BT (2010). Involvement of intracellular calcium on the phosphorylation of spinal N-methyl-d-aspartate receptor following electroacupuncture stimulation in rats. *Acta Histochemica*.

[46] Deluze C, Bosia L, Zirbs A, Chantraine A, Vischer TL (1992). Electroacupuncture in fibromyalgia: results of a controlled trial. *British Medical Journal*.

